# Editorial: Engineered neuromodulation approaches to treat neurological disorders

**DOI:** 10.3389/fnins.2022.1038215

**Published:** 2022-09-27

**Authors:** Vinícius Rosa Cota, Márcio Flávio Dutra Moraes

**Affiliations:** ^1^Rehab Technologies, Center for Convergent Technologies, Istituto Italiano di Tecnologia, Genova, Italy; ^2^Laboratory of Neuroengineering and Neurosciences, Department of Electrical Engineering, Federal University of São João Del-Rei, São João Del-Rei, Brazil; ^3^Núcleo de Neurociências, Department of Physiology and Biophysics, Federal University of Minas Gerais, Belo Horizonte, Brazil

**Keywords:** brain stimulation, neural activity, neural circuits, neurophysics, precision neuroengineering, spatial-temporal patterns

Due to its undisputed capacity of triggering brain processes with great temporal resolution (added to the fact that despite current efforts some neurological disorders are still poorly controlled), direct brain stimulation has developed into a medical and scientific field—the Neuromodulation field—with a vast constellation of efficacious therapeutic techniques and applications. Despite decades-long scientific efforts leading to knowledge advancement, the challenging complexity of the interaction between external stimuli and neurobiology has hindered the full understanding of the mechanisms underlying therapeutic effects. This jeopardizes the choice of stimulation parameters and protocols, impairs a more rational technology design, and thus has an important impact on therapeutic efficacy, efficiency, and safety. Fortunately, unprecedented development of neural interfaces and computational methods for analysis and simulation of neural systems has enabled scientists, technologists, and practitioners to carry out much-refined “precision-neuroengineering” versions of neurostimulation. This Research Topic aims to inform readers on some of the recent contributions that aid to the still ongoing effort of making the transition from historical empiricism toward rational design of techniques and technologies associated with Neuromodulation.

The neuromodulation field is very diverse, with distinct methods, targets, applications, and investigative strategies, and the papers selected for this compendium bear testimony to this statement. Experimental studies in this Research Topic were carried out *in silico* and *in vivo*, with animals and in humans, using stimuli of different physical modalities, including electrical fields, magnetic fields, and ultrasound. They have been applied to different anatomical targets, both within the central and peripheral nervous systems, in invasive, semi-invasive, and non-invasive modalities, in order to assess the effects on neurological disorders, on neural functions, or yet on general properties of neural tissue. This Research Topic includes original research papers, a case report, and a review.

Electrical stimulation directly applied to the central nervous systems, clinically termed Deep Brain Stimulation (DBS), is certainly the flagship of neuromodulation methods. Although largely studied and employed, full understanding of its therapeutic mechanisms is still lacking. Yuan et al. used hippocampal CA1 of rats in order to investigate *in vivo* the mechanisms of activity suppression induced by High-Frequency Stimulation (HFS), which is the mainstream protocol adopted in DBS. Using local field potentials and multi-unit neural electrophysiology, the authors report a series of changes in the morphology of population spikes induced by different combinations of antidromic and orthodromic axonal stimuli; which suggests an “antidromic invasion” of propagating action potentials as a possible mechanism for the sustained suppression of activity in the neuronal soma.

A major limitation of DBS is its invasiveness and the risks associated with the surgical implantation of stimulation electrodes. Collectively called Transcranial Electrical Stimulation (tES), a series of non-invasive neuromodulation techniques employ scalp electrodes for induction of electrical currents in cortical regions with distinct applications, ranging from treatment of psychiatric disorders to cognitive enhancement. Zhao et al. used a combination of such tES methods, namely Transcranial Direct Current Stimulation (tDCS) and Transcutaneous Auricular Vagus Nerve Stimulation (taVNS), as a means to improve working memory function in humans. The study not only suggests novel venues for pursuing neuromodulation clinical applications by the combination of techniques but also points to a new perspective for the understanding of the therapeutic mechanisms (i.e., by taking into consideration synergistic effects and the additive contributions of distinct anatomical targets). These thoughts resonate with several other research data suggesting that Large-Scale Integration of neuronal networks may be modulated by using appropriate spatial and temporal patterns of electrical stimulation.

Conversely, tES is criticized for its limited focality and inability to affect deeper areas of the brain. In their work, Khatoun et al. describe *in silico* evidence for a semi-invasive alternative in which a penetration effect is achieved by interferential stimulation, i.e., by simultaneously delivering electrical waveforms of slightly different frequencies and phases generated by pairs of electrodes positioned on the surface of the skull. This disruptive novel method is termed interferential epicranial electrical stimulation (IF-ECS). Using a realistic computational model of the human head, the authors carried out a finite-element computation to resolve electrical fields and investigate the focality of IF-ECS compared to conventional tES.

Other approaches in neuromodulation are carried out using non-electric physical effects as a means to deliver stimulation without any kind of contact with human tissue. This is the case of Transcranial Magnetic Stimulation (TMS) capable of generating electrical currents in the brain after electromagnetic induction imposed by external electromagnetic coils. TMS is under extensive investigation and also severe peer scrutiny. To provide evidence of therapeutic efficacy, Starnes et al. report a case of a 48-years old patient with refractory epilepsy treated acutely and in follow-ups with repetitive TMS (rTMS) together with the investigation of sleep electroencephalography (EEG) biomarkers. Robust seizure control was reportedly attained during treatment and in the ensuing months. Moreover, changes in EEG biomarkers provided evidence of rTMS effects on cortical excitability while bringing about novel insights into its mechanisms of action, including those related to brain connectivity and neural plasticity. As an aftermath, the use of TMS may also be explored as a probing stimulus for detecting abnormal responses, thus working itself as a biomarker for diagnosis and predicting neuronal instability.

Finally, Dell'Italia et al. contributed a comprehensive review on Low Intensity Focused Ultrasound (LIFU), a novel neuromodulation technique that has attracted a lot of attention recently. Of great importance, authors dedicate great effort in order to unraveling the underpinnings of its therapeutic effect by reconciling the empirical evidence with the mechanistic investigation. They review the neurophysics (in this case the Electrophysiological-Mechanical Coupling) and also the electrophysiology of LIFU to put forward the idea that neuronal intramembrane cavitation excitation (NICE) is possibly a major driving force underlying the effects of LIFU, even if throughout literature there are results that contest such findings.

Overall, the papers included in this Research Topic represent a concise but very interesting sample of the vibrant field of neuromodulation literature reporting efforts toward the development of more rational precision-tuned designs of technologies and methods. In fact, this collection brings up key messages to the field, including the undisputed value of detailed theoretical *in silico* studies (Khatoun et al.), the importance of investigating mechanisms of action in different levels of brain organization (Dell'Italia et al.; Yuan et al.), including neural plasticity-related mechanisms (Starnes et al.; Yuan et al.), and the validity of thinking outside the box in the search of novel forms of modulating the brain activity (Khatoun et al.; Dell'Italia et al.; Zhao et al.).

## Author contributions

All authors listed have made a substantial, direct, and intellectual contribution to the work and approved it for publication.

## Funding

VC was supported by the Marie Skłodowska-Curie Individual Fellowship MoRPHEUS, Grant Agreement no. 101032054, funded by the European Union under the framework programme H2020-EU.1.3. - EXCELLENT SCIENCE - Marie Skłodowska-Curie Actions. MM is a recipient of CNPq (Conselho Nacional de Desenvolvimento Científico e Tecnológico) research fellowship.

## Conflict of interest

The authors declare that the research was conducted in the absence of any commercial or financial relationships that could be construed as a potential conflict of interest.

## Publisher's note

All claims expressed in this article are solely those of the authors and do not necessarily represent those of their affiliated organizations, or those of the publisher, the editors and the reviewers. Any product that may be evaluated in this article, or claim that may be made by its manufacturer, is not guaranteed or endorsed by the publisher.

